# Understanding referral of patients with cancer in rural Ethiopia: a qualitative study

**DOI:** 10.1186/s12885-024-12294-7

**Published:** 2024-05-02

**Authors:** Josephin Trabitzsch, Morena Marquardt, Sarah Negash, Winini Belay, Yonas Abebe, Edom Seife, Kunuz Abdella, Muluken Gizaw, Sefonias Getachew, Adamu Addissie, Eva Johanna Kantelhardt, Abigiya Wondimagegnehu

**Affiliations:** 1 Global Health Working Group, Institute of Medical Epidemiology, Biometrics, and Informatics, Martin-Luther-University Halle-Wittenberg, Magdeburgerstraße 8, 06112 Halle (Saale), Germany; 2 Department of Preventive Medicine, School of Public Health, Addis Ababa University, Addis Ababa, Ethiopia; 3 Federal Ministry of Health, Addis Ababa, Ethiopia; 4 Department of Oncology, College of Health Science, Tikur Anbessa Specialized Hospital, Addis Ababa University, Addis Ababa, Ethiopia; 5 Department of Gynaecology, Martin-Luther-University Halle-Wittenberg, Halle (Saale), Germany

**Keywords:** Cancer, Healthcare system, Sub-Saharan Africa, Patient pathways

## Abstract

**Background:**

Cancer incidence is increasing in Ethiopia mainly due to increased life expectancy, while oncological capacities remain limited. Strong referral linkages between different levels of the healthcare system are key to provide timely access to cancer care. In this qualitative study, we assessed limitations and potential of cancer patient referral in the rural Southwest of Ethiopia.

**Methods:**

We held four focus group discussions (FGD) with health professionals at one primary and three secondary hospitals and conducted eight in-depth interviews (IDI) with the hospitals´ medical executives and local health bureau representatives. Data was analysed inductively using thematic analysis and emerging themes were categorized within the revised concept of access by Penchansky and Saurman.

**Results:**

The inevitable referral of patients with cancer in the rural Southwest of Ethiopia is characterized by the absence of clear communication protocols and the lack of formal referral linkages. The newly implemented hub-system has improved emergency referrals and could be expanded to non-emergency referrals, sensitive to the needs of advanced oncological care. Liaison officers can pave the way but need to be trained and equipped adequately. Referred patients struggle with inadequate transportation systems, the lack of accommodation close to specialized facilities as well as the inability to navigate at those sites due to language barriers, illiteracy, and stigmatization. Few Non-Governmental Organizations (NGOs) help but cannot compensate the limited governmental support. The shortage of medications at public hospitals leads to patients being directed to costly private pharmacies. In the light of those challenges, cancer remains to be perceived as a “death sentence” within the rural communities.

**Conclusions:**

Standardized referral linkages and a multi-faceted support network throughout the cancer care continuum are necessary to make oncology care accessible to Ethiopia´s large rural population.

**Supplementary Information:**

The online version contains supplementary material available at 10.1186/s12885-024-12294-7.

## Introduction

Referral of cancer patients between healthcare levels in Sub-Saharan Africa has often been described to be poorly coordinated, resulting in extended time to diagnosis and treatment initiation and contributing to the region´s high cancer mortality [1–3]. In 2015, Ethiopia launched its first National Cancer Control Plan aiming to reduce cancer mortality in the country by 15% by 2020. Since then, the Ethiopian government has invested heavily into oncology services, implementing prevention and screening initiatives as well as expanding diagnostic and treatment capacities. However, the availability of advanced oncology services remains limited to tertiary-level specialized hospitals located in the larger cities. Considering that 80% of the Ethiopian population are living in rural areas, strong referral linkages between rural primary and general hospitals and tertiary specialized hospitals in the cities are of particular importance to provide access to oncology care to Ethiopia´s large rural population [4, 5].

Ethiopia´s healthcare system is divided into three tiers: Health posts, health centres and primary hospitals on the lowest level, general hospitals on the secondary level and specialized hospitals on the tertiary healthcare level [1]. In terms of cancer care, at primary- and secondary-level hospitals, cancer diagnosis mostly relies on clinical findings. An affirmative diagnosis is often only possible in cooperation with pathology facilities or specialized hospitals. Primary- and secondary-level hospitals provide basic surgery for common cancers, and some offer hormonal therapy (Tamoxifen) for breast cancer patients. For advanced surgery or chemotherapy patients have to be referred to tertiary-level specialized hospitals. At time of data collection, there was only one hospital (Tikur Anbessa Specialized Hospital) providing radiotherapy.

While literature on access to oncology care in Ethiopia is increasing, referral linkages specifically have rarely been studied [6]. Furthermore, most studies addressing access to cancer care in Ethiopia have been conducted at tertiary-level cancer centres, missing out on perspectives of primary and secondary level healthcare institutions [7].

In this study we aimed to achieve a comprehensive understanding of factors influencing referral of cancer patients from primary and secondary hospitals in the rural Southwest of Ethiopia. We included healthcare professionals and representatives from local health bureaus to capture their perceptions on the existing referral system and assessed their ideas for its development in the future.

## Methods

### Study design and ethical approval

This qualitative study was conducted in December 2020 at primary and secondary hospitals in the rural Southwest of Ethiopia. It is part of a larger project aiming to design, implement, and evaluate decentralized cancer care in Ethiopia. In the writing of this manuscript we followed the Consolidated criteria for reporting qualitative research (COREQ) [8].

The study was approved by the Institutional Review Board of the Addis Ababa University College of Health Science (ref: 041/20/SPH). Study participants gave their written informed consent before each interview or discussion. All data were handled confidentially and participants´ data were pseudonymized within the transcripts.

### Participant selection and setting

The study took place at one primary and three secondary hospitals in the region of Southern Nations, Nationalities, and Peoples (Table 1).

**Table 1:** Capacity, staff, and equipment available at study sites at time of data collection (December 2020)

	Attat Our First Lady of Lourdes Catholic Primary Hospital, Wolisso	Butajira General Hospital, Butajira	Negist Elleni Mohamed Memorial Referral Hospital, Hossana	Wolaita Sodo University Teaching and Referral Hospital, Sodo
Hospital level	Primary hospital	Secondary hospital	Secondary hospital	Secondary hospital
Region	Gurage Zone	Gurage Zone	Hadiya Zone	Wolaita Zone
Catchment population	800,000	1,300,000	3,000,000	2,000,000
No. of beds	65	190	220	200
**Staff**				
No. of doctors	6	31	108	134
No. of oncologists	0	0	0	1
No. of pathologists	0	0	1	1
**Diagnostics**				
X-ray	yes	yes	yes	yes
CT	no	no	no	yes
MRI	no	no	no	on installation
FNAC	no	no	yes	yes
Biopsy	no	no	on installation	no
**Therapy**				
Chemotherapy	no	no	no	no
Hormonal Therapy	Tamoxifen	Tamoxifen	Tamoxifen	Tamoxifen
Pain medication	yes	yes	yes	yes
**Referral**				
Distance to Addis Ababa (km)	175	130	230	365
Referral hub hospital	Alert Hospital, TASH	SPMH	TASH	Hawassa Hospital, TASH

FNAC fine needle aspiration cytology;Alert Hospital Alert Comprehensive Specialized Hospital, Addis Ababa;TASH Tikur Anbessa Specialized Hospital, Addis Ababa;SPMH St. Paul´s Millenium Hospital, Addis Ababa;Hawassa Hospital Hawassa University Comprehensive Specialized Hospital

On each site, we conducted one focus group discussions (FGD) among health professionals. Participants were sampled purposefully, based on their involvement into oncology care and patient referral. Six to ten healthcare professionals participated in each FGD. In addition, we conducted in-depth interviews (IDIs) with the medical executives of each hospital, as well as with representatives from the affiliated health bureaus. Participants were approached via phone-calls prior to the interviews. All but one of the priorly arranged discussions and interviews took place; one medical executive dropped out just before the interview due to time constraints. His deputy took part in the in-depth interview instead. The FGDs took place in selected rooms within the hospital compounds while the IDIs were conducted in the participants´ private offices.

### Data collection

Discussions and interviews were conducted by two well-trained and experienced data collectors, who were one female and one male masters-level graduates. During the interviews one principal data collector acted as interviewer and moderator, while the other quietly observed the discussion taking notes.

Individualized topic guides were used to conduct interviews and focus group discussions (see Additional file 1). They were partially adapted by the data collectors to capture emerging themes as the process of data collection evolved. Interview guides were designed in English. They were translated and back translated to Amharic to ensure coherence. All interviews were conducted in Amharic. After four focus group discussions and eight in-depth interviews with key informants, data saturation was judged to have been reached. All discussions and interviews were audio-recorded, and those recordings were transcribed and translated into English by the two principal data collectors.

### Data analysis

We applied thematic framework analysis to analyse the data [9]. As suggested by Ritchie et al. we followed five analytical steps during analysis: (1) familiarisation, (2) constructing an initial framework, (3) indexing and sorting, (4) data summary and display and (5) abstraction and interpretation. We judged this to be the best suitable approach to analysis as it had been developed to be used in healthcare policy development and has since then become an often referred to approach in healthcare research. The initial framework was designed by the first author based on emerging themes and then applied parallelly by two authors to the same three transcripts. Results were discussed and the framework was adapted accordingly to ensure similar indexing and sorting. The finalized framework was then applied to all transcripts. MAXQDA 2023 was used for software support during analysis.

We used the modified “Concept of Access” to categorize our emerging findings [10, 11]. The concept was originally designed by Penchansky et al. in 1981 to describe access to healthcare within five dimensions (availability, accessibility, accommodation, affordability and acceptability) [10]. As suggested by Saurman we added awareness as a sixths dimension to mirror the total width of our data [11].

## Results

### Socio-demographics

Thirty-eight medical professionals and four health bureau representatives took part in our interviews and focus group discussions. FGDs took between 50 and 60 min, while IDIs lasted between 22 and 46 min. Participants in the FGDs were mostly nurses (15) and physicians (7). More than half of the participants had been working at the hospital for less than five years, there were 18 women and 16 men (Table 2). Participants in IDIs were predominantly male and all but one below the age of forty years. Among the medical executives were two general practitioners, one surgeon and one gynaecologist. Health bureau representatives were two non-communicable diseases focal persons, one medical service coordinator and one disease prevention team leader (Table 3).

**Table 2:** Participants of focus group discussions

	No. of participants (%) (*n* = 34)
**Interview duration** (in minutes, mean (range))	57 (50–60)
**Study site**	
Attat Primary Hospital	10 (29.4)
Butajira General Hospital	9 (26.5)
Negest Elleni Memorial Hospital	9 (26.5)
Wolayta Sodo Hospital	6 (17.6)
**Age (years)**	
< 30	18 (52.9)
30–39	10 (29.4)
40–49	4 (11.8)
≥ 50	2 (5.9)
**Gender**	
female	18 (52.9)
male	16 (47.1)
**Job title**	
nurse	11 (32.4)
physician	7 (20.6)
midwife	6 (17.6)
health officer	5 (14.7)
nurse in leadership position	4 (11.8)
pharmacist	1 (2.9)
**Working experience (years)**	
< 5	19 (55.9)
5–10	7 (20.6)
11–15	2 (5.9)
> 15	6 (17.6)

**Table 3:** Participants of in-depth interviews

	Medical executives (*n* = 4)	Health bureau representatives^a^ (*n* = 4)
**Interview duration** (in minutes, mean (range))	32 (22–45)	40 (28–46)
**Age (years)**		
< 30	1	2
30–39	2	2
40–49	0	0
≥ 50	1	0
**Gender**		
Female	1	1
Male	3	3
**Speciality**		
General practitioner	2	-
Surgery	2	-
Gynaecology and obstetrics	1	-
**Job title**		
NCD focal person	-	2
Medical service coordinator	-	1
Disease prevention team leader	-	1
**Working experience (years)**		
< 5	3	2
5–10	0	2
11–15	0	0
> 15	1	0

NCD: non communicable diseases

^**a**^ Health bureau representatives were from the following health bureaus: Gurage Zonal Health Bureau, Hadiya Zonal Health Bureau, Wolaita Sodo Zonal Health Bureau, Hawassa Regional Health Bureau.

### Perceptions on cancer patient referrals

Across all interviews and focus group discussions, participants described the need to strengthen referral linkages between primary and secondary as well as tertiary healthcare institutions. Acknowledging the major challenges referred cancer patients faced at time of this study, perceptions on the patients´ determination to overcome those barriers differed: Some participants reported that most cancer patients refused referral, while others stated patients mostly accepted the advice, eager to follow referral. Participants ideas on how to improve the referral for patients with cancer in the rural Southwest of Ethiopia are presented in the Additional file 2.

*The reason [for refusing referral] is mainly the low economical capacity of these patients and also, once they know it is cancer, they get discouraged because of what they heard about the disease. They claim that they have almost no time left to live, and it is [therefore] no use to go to the referral hospitals or it [following the referral advice] has no advantage over staying here. (FGD participant, female, nurse, age group 30–39 years)*

*It is a life and death condition for them, therefore they will go. As long as they have money, they will go. (FGD participant, female, health officer, age group 40–49 years).*

### Availability of oncology service

#### Limited oncological capacities at all levels of the healthcare system

Participants consistently identified the limited oncological capacities at all levels of the healthcare system as a major barrier to successful referral. At most sites, patients had to be referred upon suspected diagnosis or after surgery and morphological cancer verification. However, referring hospitals struggled to fulfil the receiving hospitals´ referral criteria, such as prior advanced diagnostics, that often were not available at their institution. Organizing the diagnostic workup via private diagnostic services was reported to increase costs for the patients as well as time to referral.

Regarding capacities at tertiary specialized hospitals, participants reported of patients who waited in the capital city for weeks for a preliminary appointment and were then sent back home to await their treatment initiation, which could take months.

*Sometimes they come back because the appointment for radiation is in six months, seven months or more…And they are discouraged by going up and down and I put them on my palliative care list. I just give them anti-pain. You know until then [until the reception of an appointment for treatment] they are already dead. (Hospital executive, age group ≥ 50 years)*

#### Lack of oncology specialists and training

Participants also mentioned the lack of oncology specialists at primary and secondary healthcare level. Next to the call for increased speciality and subspecialty training in oncology, health bureau representatives repeatedly mentioned the inability of rural facilities to bind trained specialists. Medical staff and health bureau representatives alike experienced that once trained adequately, oncology specialists would move to the larger cities and specialized cancer centres. Participants suggested to address the lack of oncological expertise in rural areas by establishing formal teachings from specialists to healthcare professionals from lower-level hospitals.

### Accessibility

#### Road infrastructure and transport

Due to the limited number of cancer centres throughout the country, referred patients often had to travel far to receive adequate treatment. Ethiopia´s road infrastructure was perceived to challenge patients substantially, hindering them to receive services and travel back home in one day.

Most cancer cases were non-emergency referrals and therefore patients had to organize their own transport. Besides the associated costs, patients weakened by the disease and associated treatment often were too sick to endure travelling with public transport.

### Accommodation

#### Protocols and communication within referring hospitals

In all settings, medical seniors were the ones deciding to refer patients. They would also be the ones writing the referral letter or communicating with the liaisons´ office in case of emergencies. Even though health bureau representatives highlighted the sites´ obligation to have appropriate protocols in place, formal referral protocols were only implemented at one site.

In two FGDs, the topic of unclear communication among professionals within the hospital emerged. This could lead to different parties making differing referral arrangements and resulted in patients being referred inadequately.

#### Liaisons and the hub-system for emergency referrals

At all sites, emergency referrals were organized via the formal liaison system. Nurses designated as liaisons were in charge of calling the receiving hospital in advance to check for availability of beds and announce the patients´ arrival. Even though participants agreed that the implementation of liaisons had brought major improvements to the system of emergency referrals, shortcomings of the system were discussed across all interviews and focus group discussions:

Liaisons, both at the referring as well as at the receiving institution, were perceived as not well trained to fulfil their responsibilities. They often did not grasp the urgency of the referral, or in case of cancer patient referrals, would refer patients to sites that did not offer cancer treatment.

Another perceived barrier to efficient referral of cancer patients was the newly implemented “hub system”. All primary and secondary hospitals had predefined “hub hospitals”, which were tertiary specialized hospitals acting as the first site to address when referring emergencies to a higher level. However, many of those hub hospitals did not offer oncology services, resulting in referring hospitals omitting the hub system to find adequate care for their patients with cancer. However, patients would often be rejected from hospitals other than the official hub hospital. One positive aspect of the system was the establishment of command posts: Those were interposed, site-independent units facilitating emergency referrals from lower-level hospitals to specialized facilities. Their implementation was perceived to have smoothened communication between referring and receiving hospitals.

Most of the cancer referrals at the study sites were non-emergencies. The extent to which the liaisons´ office was involved into their organization differed between hospitals. Two sites did not involve liaisons into non-emergency referrals at all. Referral letters were written by a senior doctor and patients had to organize their travels by themselves. At other sites referral letters written by doctors had to be signed and registered by the liaisons office who then supported patients in organizing their travels. In some cases, prior phone-calls were made by the health professionals to receiving institutions checking for capacities. However, this was not the norm. Participants consented that non-emergency referral via a well-equipped liaisons office, with the referring site checking for capacity and communicating the referral to the receiving site, would have a substantial impact on cancer patient referral.

#### Data management

Data management was perceived to play another important role in cancer patient referral. At time of data collection, patients carrying a referral letter were often the only form of communication between hospitals. However, professionals generally judged hand-written referral letters to be an unreliable source of data transmission. Participants from one hospital reported, that the strengthening of the liaison system had improved referral writing practices at their institution, because liaisons would no longer accept imprecise entries on the referral letter. Furthermore, there was a collective call for digital data management between hospitals, allowing for the transfer of patient data.

Participants also hoped that a digital form of data management would improve the feedback system regarding referred patients. Even though health bureau representatives described an official feedback system to be in place, currently hospitals only heard back from referred patients, when they had relatives working in the hospitals or if patients came back for further treatment. Health bureaus stated to be aware of the problem regarding the management of data and highlighted the governments ambitions to improve data quality in health facilities.

*If you would only know how people for example get a chance for radiation. Like a feed-back of how meaningful this referral was, whether it makes sense to refer people or whether they are just spending their money and time for nothing…so just simple feed-back on what is possible and what is not possible, would be nice. (Hospital executive, age group ≥ 50 years)*

#### Communication between hospitals

Poor communication between primary- and secondary-level hospitals on one side and tertiary hospitals on the other side was consistently lamented. The formal line of communication via liaisons offices was described to be unreliable due to lack of equipment and congestion.

Informal communication on the other side mostly relied on personal connections between health professionals: At referring sites where professionals knew staff from receiving sites personally, communication worked better than at sites without personal connection.

Besides communication, participants repeatedly regretted the lack of a formal way of receiving updates on services offered at receiving institution. For instance, one medical director did not know about a second hospital having recently started chemotherapy in the capital city. Health bureau representatives confirmed this observation, adding that even in health bureaus they often were not up-to-date regarding services being offered in their catchment area. As a result, patients were referred to hospitals with inadequate treatment capacities.

In the absence of well-functioning formal referral linkages, at some sites a non-governmental cancer organisation acted as the major link between the hospitals. They ensured patients met all the requirements for acceptance at the receiving institution and communicated with receiving hospitals about the referred patients. Multiple participants suggested panel discussions with members from all healthcare levels to establish personal connections and improve communication between hospitals.

*And nationally, it would be better if there was a forum prepared for hospitals to exchange their experience and discuss ways to ease treatment for referral patients. For example, a forum between our hospital and TASH [Tikur Anbessa Specialized Hospital]. I believe the health system has many stakeholders such as government organizations, government bodies and so on, so there needs to be a regular forum which includes all the stakeholders. (FGD participant, female, midwife, age group < 30)*

#### Patient navigation

Across all interviews and discussions participants highlighted, that patients with cancer were often severely ill and therefore needed much support with the facilitation of their referral. Existing projects proving the success of “patient navigators” assisting patients throughout their pathways were discussed: At one hospital site a “cancer nurse” was responsible for accompanying patients with cervical cancer to the receiving institutions. Other participants reported of an NGO providing similar services for patients with cancer. Besides establishing skilled hospital personnel or volunteers from NGOs as patient navigators, one participant suggested the introduction of so called “case managers” (former cancer patients) accompanying patients throughout their journey. In the past, this approach had proven to be successful in the context of the referral of patients with HIV.

*If the patient links with Mathiwos Wondu Ethiopian Cancer Society then things going smoothly because they receive the patient [in Addis Ababa] and facilitate processes, including cost coverage. But this association cannot reach to all cancer patients, so it is better to expand such kind of program. (Health bureau representative, age group 30–39)*

#### Reception at receiving institutions

Finding the correct services at the receiving institution could be a challenge to referred patients. Many patients were not able to read and could therefore not follow signs at the receiving institution. Furthermore, some patients did not speak Amharic, the language spoken in the capital city.

At the time of the study, patients that were not referred by ambulance had to go through the receiving hospital´s OPD before getting connected to the oncology unit. A fast-track system, channelling referred patients with cancer directly to the oncology unit was thought to decrease waiting times at the receiving institution.

*Finding the unit at the hospital compound is also other challenge. I heard there was a patient who came back from the hospital, where he had been referred to, without getting the investigation and management, because he did not find the exact room. So, it’s better to modernize the reception and assign individuals to show the way to the units to which they [the patients] were referred to. (Hospital executive, age group < 30 years)*

### Affordability

#### Patients handling of costs

Participants discussed how the patients’ economic status directly affected their ability to follow the referral advice. It determined not only where they would be referred to (more costly private institutions offered treatment with substantially shorter waiting times), but also whether they could follow the referral at all. Healthcare workers emphasized the efforts patients made to comply with the referral. Because of the disease, patients often were unable to work and cover the costs themselves. Mostly, costs were arranged with the family or within the communities. Some patients rented their land or sold their property. However, facing the uncertainty of surviving the disease even if they followed the referral, many patients refused referral. Those patients then turned to cultural healers or went back home, possibly preparing for death.

#### Governmental support

In the absence of universal health insurance, the Ethiopian government provides free cancer treatment at public hospitals for patients with a low economic background. However, participants reported regular shortages of medications at tertiary public hospitals. In those occasions, even patients eligible for free treatment were told to buy medication at private pharmacies – leading to them having to pay for treatment which was supposed to be free. In addition, the governments´ support did not cover expenses for food and accommodation before and during the treatment. Patients relied on having relatives in the city. Participants agreed that those factors often resulted in patients not being able to afford following the referral advice, even though they were eligible for free treatment.

#### Non-governmental support

Compensating for the lack of governmental aid, all study sites had support mechanisms in place trying to enable patients financially to follow the referral advice. Those mechanisms included social workers assisting the patients in raising the necessary money as well as the provision of free transport. However, the hospital´s ability to support was perceived to be insufficient, often resulting in staff personally donating money for patient referrals. Again, NGOs were also perceived to play an important role in decreasing direct and indirect costs for patients. They paid for travel expenses and offered food and accommodation in staying houses close to the receiving hospitals.

### Awareness

#### Health education

There was large consent on the importance of educating patients about their cancer disease. Participants highlighted, that patients would only follow the referral advice, if they were informed properly about their disease and its possible outcomes. The provision of health education was described to take place on different levels: On community-level (health extension workers, public gatherings, and mass media), hospital-level (lectures on health-related topics in the waiting areas each morning) as well as on a one-on-one level during appointments. There was a general perception, that educated and well-informed patients were more likely to follow the referral advice. Uneducated and uninformed patients would often turn to traditional medicine instead.

*Most of the time people tend to do what they believe. So, if they understand well, they don’t hesitate to follow a referral process unless they may have financial problem. (Hospital executive, age group < 30 years)*

#### Availability of public information on services

In addition to information about the cancer disease, participants agreed on the importance of providing patients with information on costs and waiting times. They regretted not being able to give patients the numbers on how much money they needed at the receiving institution. This led to referred patients having to return home without any treatment, because they ran out of money while waiting.

### Acceptability

#### Trust in the health system

The patients´ perception of referral as a “death sentence” was one more emerging theme. Even though, knowledge about cancer in rural communities was perceived to have increased over the last years, cancer was still reported to have a fatal reputation. Rumors of rejection and long waiting times at the specialized hospitals added to the patients´ believe that once referred, they would never come back alive.

Healthcare professionals suggested to learn from the countries´ experiences with HIV which also used to be perceived as “death sentence”. Participants suggested to extend existing cancer awareness campaigns and include education about cancer into the curriculum of health extension workers. Establishing cancer as a treatable disease in communities and families was felt to be essential to convince patients of the significance of following the referral.

*So likewise, when we come to cancer, it follows a similar pattern as when HIV first came to Ethiopia. HIV patients used to feel hopeless and likewise cancer patients are feeling like that now. Secondly, the status of HIV reached what it has today due to integrated relent less effort, so if we do the same with cancer, I bet we could save lots of lives. (FGD participant, female, nurse, age group 40–49 years)*

#### Stigmatization

One more barrier to successful referral was reported to be the stigmatization patients from rural areas experienced at tertiary hospitals. Patients often returned to referring hospitals complaining of having been handled badly at the receiving institution. Establishing a welcoming environment at receiving hospitals was regarded to contribute to the patients´ successful treatment initiation at the tertiary healthcare level.

*They [referred cancer patients] come [back] and complain “We would rather die here than go there [again], they don’t accept us as you do, they don’t talk to us as you do, they harass us saying ‘people who come from rural areas’, the place they give [us] is not good.’ They also complain like ‘they host those who come from near that area, and they only give distant appointments to us.’ (FGD participant, female, nurse in a leadership position, age group < 30)*.

## Discussion

Patients with cancer referred from primary and secondary hospitals to tertiary specialized care face challenges in all dimensions of Penchansky´s revised concept of access. We found a broad range of experiences among medical and local health bureau representatives on how to address those challenges aiming to provide access to cancer care in rural Ethiopia.

Participants felt strongly about the need to increase oncological capacities throughout the country as a foundation for successful referral. Expanding specialized training and connecting specialists in oncological societies are tools to strengthen a nation´s expertise in oncology care [12]. Ethiopia´s first clinical oncology residency program was initiated at Tikur Anbessa Hospital in 2013. Since 2019, the “Ethiopian Society of Haemato-Oncology” serves as a national platform for knowledge exchange. One intervention suggested by participants of the study was the training of non-oncology-specialists to provide oncological diagnostics and treatment. Experiences with this task shifting model from other countries in Sub-Saharan Africa are promising: An example could be the training of non-oncologists (internists, paediatricians, general practitioners, and nurses) in delivering cancer care, as it has been successfully done in Rwanda over the past year [13].

We identified three possible ways patients with cancer were referred: non-emergency referral via referral letter only, non-emergency referral via the liaisons´ office and emergency referrals. Most cancer patients were non-emergencies and mostly referred via referral letters only. Standardized referral letters had been introduced by the Ministry of Health in 2010 and were used in all four hospitals [14].

While oncological emergency referrals were organized via the official hub-system, for non-emergency referrals there were no inter-hospital referral protocols. An intra-institutional protocol for cancer patient referral was in place at only one of our study sites. Knowledge on where to refer patients with cancer relied on the health professionals´ personal experiences and expertise. Even though the hub-system for emergency referrals was perceived to be a barrier to the referral of patients with cancer, participants consented, that having a set system defining where to refer patients had improved emergency referrals in general. Expanding the system to include non-emergency referrals, sensitive to the availability of advanced oncological care, could improve referral of patient with cancer substantially.

In its “Guideline for implementation of a patient referral system” the Ethiopian Ministry of Health foresaw a “referral coordinator” at each hospital - responsible for the facilitation of emergency as well as non-emergency referrals [14]. In our study, only one hospital reported organizing non-emergency referrals via the liaisons´ office (which served as referral coordinator). Training and equipping designated personnel at the liaisons´ office, enabling them to organize both emergency and non-emergency referrals in a reliable manner emerged to be of substantial significance when improving cancer patient referral.

An important aspect in strengthening the referral linkage between healthcare levels is the standardization of data management. At the time of our study, patient data was collected in paper files and referral-letters were hand-written. Currently the Ministry of Health is developing a standardized electronic health record system to “strengthen digitization of routine and non-routine data collection, management, analysis and use” [15].

To enable patients to follow referral advice, participants also suggested the implementation of patient navigators. In high-income countries patient navigation programs are a well-established tool to promote access to cancer care [16]. Nurses or lay persons, who have been trained to be “patient navigators”, accompany patients with cancer throughout their diagnostic and treatment journey. Depending on the extent of the program, patient navigators provide patients with health education, facilitate appointments, and arrange linkages to follow-up services. In countries without universal health coverage, the navigation services often also include stipends for transport, accommodation, and treatment [17, 18]. In the past years, the concept of patient navigation has been increasingly adapted in low- and middle-income countries, showing positive effects on outcomes like treatment initiation and adherence [19]. Initiatives such as the BEACON Initiative (Building expertise, advocacy, and capacity for oncology navigation) launched by the American Cancer Society implementing patient navigation programs at national referral hospitals in Uganda and Kenya serve as examples [20].

Furthermore, a smooth reception of referred patients at the receiving institutions was identified to be key for a successful referral. Participants suggested to address the patients´ difficulties in finding the correct units at the receiving hospitals by establishing an easy-to-follow signposting, as well as staffed info-points. Such low-cost concepts, sensitive to the patients´ different language and educational backgrounds, have previously shown to improve the patients´ experiences at receiving institutions substantially [21].

A current project addressing the need for “fast-track” pathways for patients with cancer at receiving institutions is the “Walk-in-Clinic” at the Else-Kroener-Center for Cancer Care in Addis Ababa. In collaboration with the surgical and oncology units at Tikur Anbessa Hospital the centre enables women with suspected cancer to omit the usual out-patient department pathways and be directly seen by gynaeco-oncologists and breast surgeons [22].

Direct and indirect costs were perceived to be a major barrier to successful referral. Patients with sufficient financial means could visit private hospitals or receive care in countries with better access to high-quality healthcare. However, with an income per capita of 940$ and approximately one quarter of the population living below the international poverty line, most patients in Ethiopia rely on the public healthcare sector [23, 24]. Ethiopia aims to establish a universal health coverage by 2030 [1]. To reach this ambitious goal, the government has established multiple channels to increase access to healthcare for its population: Social health insurance is currently being implemented for people working in the formal sector, while community-based health insurance schemes are successfully expanded within the large informal sector [1]. In addition, certain oncology-associated interventions were recently added to the “Essential Health Services Package”, guaranteeing their provision free of charge, or with cost-sharing and cost-recovery mechanisms in place at public hospitals [25]. Patients who cannot afford care are eligible for free treatment, provided they receive a “fee waiver” from their local health bureau [1, 26].

Nevertheless, the multitude of unofficial financial support mechanisms in place at all study sites demonstrates that patients with cancer in rural Ethiopia still face substantial financial challenges. Unavailability of necessary medications in public institutions (resulting in patients having to buy treatment at private pharmacies) as well as the costs for transport and accommodation associated with the treatment, emerged to be major barriers to oncology care. These findings are confirmed by previous studies [26–29]. Reliable mechanisms for the procurement and financing of cancer drugs at public hospitals are needed to decrease direct costs of treatment. Indirect costs could be tackled by the provision of governmental travel stipends for those in need as well as the establishment of staying houses close to the cancer centres.

We found high awareness regarding the significance of health education in the provision of cancer care among health professionals and health bureau representatives. This mirrors the governments´ focus on health education since the launch of Ethiopia´s Health Extension Program in 2003. Core of the program are health extension workers who promote primary healthcare on the community level. A study by Funga et al. found health extension workers to be the main source of information on cancer for most of the rural population [30]. However, even though the Health Extension Program has proven to be highly successful in providing health education to Ethiopia´s rural population, awareness on cancer is still insufficient and the perception of cancer as a “death sentence” common [30–33]. Expansion of existing and initiation of new cancer awareness programs is therefore essential to increase knowledge about cancer in rural communities. In addition, the implementation of survivor groups could contribute to change the patients´ attitude towards cancer and improve trust into the healthcare system [34]. Establishing cancer as a curable disease is important to convince patients to follow the referral advice.

Interestingly, while much reported previously in the context of access to cancer care, stigmatization of patients in their community did not emerge to be a major barrier to successful referral in our study [35]. This might be explained by the healthcare professionals´ and health bureau representatives´ perspective of the study. A recent study on the perceptions of cervical cancer care among Ethiopian women and their providers supports this explanation: While patients discussed the role of stigmatization within their communities vividly, providers did not mention stigma as a major barrier to care [35]. On the other hand, participants did report about patients feeling stigmatized at receiving institutions due to their rural background. While we could not find any literature on stigmatization of rural patients in specialized hospitals in Ethiopia, poor handling and disrespectful communication at tertiary hospitals has been described before [35, 36]. Further research on patients´ experiences at tertiary hospitals as well as health professionals´ training is needed to guarantee culturally sensitive access to cancer care.

We believe it a great strength of this study to have captured a broad variety of perspectives of healthcare professionals and health bureau representatives who are involved into cancer care at the primary and secondary level of the healthcare system. However, our study has certain limitations: First and foremost, our results are limited by the participants´ provider perspective. To receive a comprehensive understanding of the cancer patient referral in rural Ethiopia, perspectives of referred patients have to be considered. In terms of sample size, the number of IDIs and FGDs conducted for this study falls within the lower end of what is typically considered adequate in qualitative research. However, while we cannot exclude the possibility that an increased sample size would have contributed new data, after four FGDs and eight IDIs we felt we had reached data saturation. Working with a small sample size increases the importance of thorough purposive sampling. Following the guidance of Ritchie et al., we ensured best possible representation and diversity within the sample with regards to variables such as age, years of experience, field of expertise, healthcare level, and hospital size [9]. This approach also helped to minimize, however not eliminate, a potential sampling bias.

Furthermore, we did not use a formal protocol regarding the triangulation of data collected by different methods and from different participant groups. However, in the final phases of analysis we did colour-code different origins of elements within themes and subthemes to be aware of consent and contradictions between the different participant groups.

## Conclusions

In the rural Southwest of Ethiopia, decision makers are aware of multi-factorial challenges cancer patients face when being referred from lower-level hospitals to tertiary-level oncology care. A way forward requires a multi-faceted approach involving capacity building, improved coordination between different levels of the healthcare system, standardized protocols and data management, financial and social support mechanisms, as well as awareness programs. Lay persons as patient navigators could be involved. Establishing an environment for inter-institutional exchange and integrating stakeholders´ broad experiences from the primary and secondary healthcare level into future policy making is a key to reduce disparities in cancer care and make oncology care available to Ethiopia´s large rural population.

### Electronic supplementary material

Below is the link to the electronic supplementary material.

Supplementary Material 1

Supplementary Material 2

## Data Availability

The datasets used and analysed during the presented study are available from the corresponding author on reasonable request.

## Abbreviations

COREQ: Consolidated criteria for reporting qualitative research
FGD: focus group discussion
IDI: in-depth interview
NGO: non-governmental organization
OPD: outpatient department
FNAC: fine needle aspiration cytology
NCD: non-communicable diseases
