# Supplementary material for: Understanding referral of patients with cancer in rural Ethiopia: a qualitative study
Source: BMC Cancer. 2024 May 2;24:553. doi: 10.1186/s12885-024-12294-7 (PMC11067183; doi:10.1186/s12885-024-12294-7)
Supplement: Supplementary file 1 — Supplementary Material 1 [file 12885_2024_12294_MOESM1_ESM.docx]

### Additional File 1 | Topic and Interview Guides

### Focus group discussions with health professionals

| Q1 | To begin with, could you please help me understand the reasons, why cancer patients are usually referred from your hospital to other hospitals? | 5 min |
| --- | --- | --- |
| Q2 | Could you describe, how the process of referral of cancer patients is organized in your hospital? | 10 min |
|  | - Who refers patients? - Where are patients usually referred to from here? - Are there referral guidelines for cancer patients in your hospital? - Which information will patients receive when referred? Do they get contact names or numbers? - Do you personally know the doctors you refer patients to? - Are patients usually referred with a specific objective? - Do you usually hear back from patients, who you have referred? - Have there been efforts to improve the referral system in the past? |  |
| Q3 | In your experience, how is this process of referral working in the moment? | 10 min |
|  | - Which aspects of the referral process are working well? - Which aspects of the referral process aren´t working so well? - Which challenges do patients face when they are being referred for further care? - Are there aspects in the referral process/system are today better compared to in the past? - Which aspects in the referral process/system are worse compared to in the past? |  |
| Q4 | How much are patients being involved in the decision of referral? | 10 min |
|  | - How long would a talk about a possible referral usually last? - How do you determine whether patients plan to follow your advice of referral? - Which alternatives of treatment are offered, when patients state, not being able to follow referral advice? |  |
| Q5 | In your opinion, which actions need to be undertaken to improve the referral process of cancer patients between secondary and tertiary care? | 10 min |
|  | - Which changes in the health system are needed? - What should policy makers do? - Which research is needed? - Which actions must be undertaken on hospital level? Who is responsible? - Which measures can you personally take? What would you need to participate in improving the referral system? |  |

### In-depth interviews with medical executives

| Q1 | To begin with, could you explain your area of expertise and your responsibilities in this hospital? | 5 min |
| --- | --- | --- |
| Q2 | Could you please explain, how referral of cancer patients is organized at this hospital? | 10 min |
|  | - What are usually the indications for referral of cancer patients? - Which information do you need, to make the decision of referral? - Are there referral guidelines for cancer patients in your hospital? - Are patients usually referred with a specific objective? - Do you hear back from patients, who you have referred? - Which parties will be involved into the referral process (doctors, nurses, social workers etc.? |  |
| Q3 | What would a typical talk with a cancer patient about his referral look like? | 5 min |
|  | - What do you usually tell patients, why they are being referred? - Which information will patients receive when referred? Do they get contact names or numbers? - What happens, when a patient tells you, he or she won´t be able to follow your referral advice? - Which main challenges in being referred are being communicated to you by the patients? |  |
| Q4 | How would you describe the hospitals relationship with the referral hospitals? | 5 min |
|  | - Which hospitals do you usually refer to? - Do you personally know doctors at the hospitals you refer patients to? - Which form of communication is there between hospitals? |  |
| Q5 | In your opinion, how does the referral of cancer patients work at this point? | 10 min |
|  | - Which aspects of the referral process are working well? - Which aspects of the referral process aren´t working so well? - In your view, which challenges to patients face after referral? - In your experience, which factors influence the referral of patients positively? |  |
| Q6 | In your opinion, which actions need to be undertaken to improve the referral process of cancer patients within secondary and tertiary care? | 10 min |
|  | - Which changes in the health system are needed? - What should policy makers do? - Which research is needed? - Which actions must be undertaken on hospital level? Who is responsible? - Which measures can you personally take? What would you need to participate in improving the referral system? |  |

### In-depth interviews with health bureau representatives

| Q1 | Let us start by you telling me about you, your profession, and your responsibilities in this institution. | 5 min |
| --- | --- | --- |
| Q2 | How would you describe your institution´s relationship with the hospitals in your area? | 5 min |
|  | - What form of communication is there? What level of exchange is there? - Concerning which topics will you be addressed from regional hospitals? - Do you feel like, you are aware of the challenges, regional hospitals are facing? |  |
| Q3 | Which role does cancer care play in your work? | 5 min |
|  | - To what extent have you previously been involved in cancer care? - Which actions are there currently addressing cancer care? - What do you see as the biggest challenges in cancer care? |  |
| Q4 | What comes to your mind when you hear the term “cancer patient referral”? | 5 min |
|  | - How is referral between hospitals organized in your region? - Which examples for well working referral processes between hospitals come to your mind? - Have there been actions targeting patient referral in the past? - Which aspects do you think facilitate the referral system today as compared to 5 years ago? - Which aspects make the referral system more difficult today as compared to 5 years ago? |  |
| Q5 | Which patient mediated factors play a role in successful cancer patient referral? | 5 min |
|  | *(e.g. education, traditional believes, socioeconomic status)* |  |
| Q6 | Which health system mediated factors play a role in successful cancer referral? | 5 min |
|  | *(e.g. education of doctors, communication between hospitals, infrastructure, capacity building)* |  |
| Q7 | In your opinion, how can referral of patients between hospitals be improved in order achieve timely diagnosis and treatment of cancer patients? | 5 min |
|  | - Actions on regional hospital level (referral guidelines, information, communication) - Actions on referral hospital level (capacity, coordination, information) - Action on governmental level? (infrastructure, policies) |  |
| Q8 | Which steps could specifically your institution undertake, to improve cancer patient referral? | 5 min |
