# Supplementary material for: Understanding referral of patients with cancer in rural Ethiopia: a qualitative study
Source: BMC Cancer. 2024 May 2;24:553. doi: 10.1186/s12885-024-12294-7 (PMC11067183; doi:10.1186/s12885-024-12294-7)
Supplement: Supplementary file 2 — Supplementary Material 2 [file 12885_2024_12294_MOESM2_ESM.docx]

### Additional File 2 | Health professionals´ and health bureau representatives´ ideas on how to improve access to oncology care for patients with cancer diagnosed at primary and secondary-level hospitals in the rural Southwest of Ethiopia.

|  | **Short-term approaches**  (1 – 2 years) | **Medium-term approaches**  (3 – 5 years) | **Long-term approaches**  (6 – 10 years) |
| --- | --- | --- | --- |
| **AVAILABILITY** |  |  |  |
| Exceeded oncological capacities at all levels of the healthcare system | - Standardize public-private partnerships for oncological diagnostics at primary and secondary level of the healthcare system. - Establish instruments to maintain and repair existing capacities. - Establish palliative care for patients unable to be referred at all levels of the healthcare system. | - Expand diagnostic capacities on the primary and secondary healthcare level to enable diagnostic workup before referral. - Expand number of beds on the tertiary healthcare level. - Establish reliable sources of oncology-associated supplies on all healthcare levels. | - Expand on a regional level diagnostic and treatment capacities on all levels of the healthcare system. |
| Lack of oncology specialists and training | - Establish specialists´ support at primary and secondary hospitals (video conferences, e-learning). | - Oncology trainings from specialists to general practicioners (task shifting model) - Establish incentives for skilled personnel to work outside of the capital city. | - Expand clinical oncology and other associated residency programs at universities throughout the country. |
| **ACCESSIBILITY** |  |  |  |
| Road infrastructure and transport | - Establish assistance in the organization of travels at primary and secondary hospitals. | - Increase number of ambulances. - Provide travel stipends for non-emergency referrals. | - Improve road-infrastructure and public transport in rural Ethiopia. |
| **ACCOMMODATION** |  |  |  |
| Protocols and communication within referring hospitals | - Introduce protocols for referral of cancer patients at primary and secondary hospitals. - Define clear responsibilities in primary and secondary hospitals regarding referral of cancer patients. - Implement interdisciplinary monitoring and evaluation panels regarding patient referral within referring hospitals. |  |  |
| Liaisons and the hub-system for emergency referrals | - Equip liaisons at all healthcare levels with necessary hard- and software. - Adapt hub-system to be flexible to the needs of cancer patient referrals. | - Train liaisons regarding the demands of cancer patient referral. - Increase liaisons capacities to coordinate emergency and non-emergency referrals. - Establish “bed occupancy manager”, checking for availability of beds before referral. |  |
| Data management | - Establish teachings regarding the importance of complete and readable information on referral letters at primary and secondary level hospitals. | - Digitalize data handling and storage on institutional level. - Enable printing of referral letters to ensure readability of information. | - Establish digitalization of inter-institutional data handling and transfer. - Establish digital feedback-system on inter-institutional referrals. |
| Communication between hospitals | - Define reliable lines of communication between institutions. - Establish reliable channels of information regarding updates on care provision in referral hospitals. | - Establish regular platforms for exchange between different-level healthcare institutions (inter-level panel discussions) |  |
| Patient navigation | - Engage local patient navigators at receiving hospitals. | - Train patient navigators at referring hospitals (cancer survivors, volunteers, health professionals) |  |
| Reception at the receiving institution | - Design easy-to-follow signposts at receiving institutions. - Expand information posts at receiving institution to assist referred patients in finding the correct rooms. | - Establish easily accessible translator services at receiving institutions. | - Implement fast-track system for referred cancer patients at the oncology department of receiving institutions. |
| **AFFORDABILITY** |  |  |  |
| Governmental support | - Offer stipends for travel and accommodation in addition to fee waivers for eligible patients. | - Establish accommodation, “staying houses”, for patients close to receiving institutions. - Establish reliable supply channels of cancer medication at public hospitals. | - Expand social health insurance and community-based-health insurance. |
| Non-governmental support |  | - Integrate NGO´s experiences and potential into future policy making. |  |
| **AWARENESS** |  |  |  |
| Health education | - Involve family (main source of support) into medical consultations and educational talks at referring institutions. - Establish health education of patients diagnosed with cancer at referring institutions. | - Expand awareness campaigns on a community level. - Expand extent of education about cancer in Health Extension Workers´ curriculum. |  |
| Availability of public information on services | - Collect data on costs and waiting time to be expected by patients when following referral advice to tertiary-level specialized care. |  |  |
| **ACCEPTABILITY** |  |  |  |
| Trust in the health system | - Establish cancer survivor groups on a community-level. | - Establish peer-counseling, were cancer survivors support patients throughout their journey. |  |
| Stigmatization | - Collect data on stigmatization of rural patients with cancer in receiving hospitals. | - Implement trainings of health care professionals at receiving institutions regarding culturally sensitive handling of patients. |  |
